# Associations Between Cognition and Serotonin 1B Receptor Availability in Healthy Volunteers: A [^11^C]AZ10419369 Positron Emission Tomography Study

**DOI:** 10.1093/ijnp/pyac084

**Published:** 2022-12-27

**Authors:** Ämma Tangen, Emma R Veldman, Jonas Svensson, Mikael Tiger, Magdalena Nord, Kimmo Sorjonen, Max Andersson, Pontus Plavén-Sigray, Andrea Varrone, Christer Halldin, Katarina Varnäs, Jacqueline Borg, Johan Lundberg

**Affiliations:** Centre for Psychiatry Research, Department of Clinical Neuroscience, Karolinska Institutet, & Stockholm Health Care Services, Stockholm County Council, Stockholm, Sweden; Centre for Psychiatry Research, Department of Clinical Neuroscience, Karolinska Institutet, & Stockholm Health Care Services, Stockholm County Council, Stockholm, Sweden; Centre for Psychiatry Research, Department of Clinical Neuroscience, Karolinska Institutet, & Stockholm Health Care Services, Stockholm County Council, Stockholm, Sweden; Neurobiology Research Unit, Copenhagen University Hospital, Copenhagen, Denmark; Centre for Psychiatry Research, Department of Clinical Neuroscience, Karolinska Institutet, & Stockholm Health Care Services, Stockholm County Council, Stockholm, Sweden; Centre for Psychiatry Research, Department of Clinical Neuroscience, Karolinska Institutet, & Stockholm Health Care Services, Stockholm County Council, Stockholm, Sweden; Division of Psychology, Department of Clinical Neuroscience, Karolinska Institutet, Stockholm, Sweden; Centre for Psychiatry Research, Department of Clinical Neuroscience, Karolinska Institutet, & Stockholm Health Care Services, Stockholm County Council, Stockholm, Sweden; Centre for Psychiatry Research, Department of Clinical Neuroscience, Karolinska Institutet, & Stockholm Health Care Services, Stockholm County Council, Stockholm, Sweden; Neurobiology Research Unit, Copenhagen University Hospital, Copenhagen, Denmark; Centre for Psychiatry Research, Department of Clinical Neuroscience, Karolinska Institutet, & Stockholm Health Care Services, Stockholm County Council, Stockholm, Sweden; Centre for Psychiatry Research, Department of Clinical Neuroscience, Karolinska Institutet, & Stockholm Health Care Services, Stockholm County Council, Stockholm, Sweden; Centre for Psychiatry Research, Department of Clinical Neuroscience, Karolinska Institutet, & Stockholm Health Care Services, Stockholm County Council, Stockholm, Sweden; Centre for Psychiatry Research, Department of Clinical Neuroscience, Karolinska Institutet, & Stockholm Health Care Services, Stockholm County Council, Stockholm, Sweden; Centre for Psychiatry Research, Department of Clinical Neuroscience, Karolinska Institutet, & Stockholm Health Care Services, Stockholm County Council, Stockholm, Sweden

**Keywords:** 5-HT_1B_, cognition, healthy volunteers, positron emission tomography, serotonin

## Abstract

**Background:**

The serotonin system has been implicated in several psychiatric disorders. All major psychiatric disorders are associated with cognitive impairment, but treatment improving cognitive deficits is lacking, partly due to limited understanding of the neurobiology of cognitive functioning. Several markers for the serotonin system have been associated with cognitive functions. Our research group previously has reported a positive correlation between serotonin (5-HT_1B_) receptor availability in the dorsal brainstem and visuospatial memory in a pilot study of healthy individuals. Here, we aim to replicate our previous finding in a larger group of healthy volunteers as well as to investigate putative associations between 5-HT_1B_ receptor availability and other cognitive domains.

**Methods:**

Forty-three healthy individuals were examined with positron emission tomography using the 5-HT_1B_ receptor radioligand [^11^C]AZ10419369 and a visuospatial memory test to replicate our previous finding as well as tests of verbal fluency, cognitive flexibility, reaction time, and planning ability to explore other domains potentially associated with the serotonin system.

**Results:**

Replication analysis revealed no statistically significant association between 5-HT_1B_ receptor availability in the dorsal brainstem and visuospatial memory performance. Exploratory analyses showed age-adjusted correlations between 5-HT_1B_ receptor availability in whole brain gray matter and specific brain regions, and number of commission errors, reaction time, and planning ability.

**Conclusions:**

Higher 5-HT_1B_ receptor availability was associated with more false-positive responses and faster reaction time but lower performance in planning and problem-solving. These results corroborate previous research supporting an important role of the serotonin system in impulsive behavior and planning ability.

**Significance Statement**

The brain serotonin system has been implicated in several psychiatric disorders. All major psychiatric disorders are associated with cognitive impairment, but treatment improving cognitive deficits is lacking. This study aimed to improve knowledge of the neurobiological underpinnings of cognition by replicating previous findings of a positive association between 5-HT_1B_ receptor availability and visuospatial memory and exploring additional associations between 5-HT_1B_ receptor and cognition in healthy individuals. Exploratory analyses demonstrated correlations between 5-HT_1B_ receptor availability in whole brain gray matter and several specific brain regions, and number of false-positive responses, reaction time, and planning ability. The replication analysis revealed no significant association. In conclusion, although we did not replicate our previous finding of an association between 5-HT_1B_ receptor availability and visuospatial memory, our results corroborate previous research supporting an important role of the central serotonin system in planning ability and impulsive behavior.

## INTRODUCTION

All major psychiatric disorders are associated with cognitive impairment, symptoms that often fail to respond to existing treatments and commonly linger long after core features of the disorder are in remission. Limited understanding of the neurobiological underpinning of cognitive functioning has hampered the development of treatments specifically targeting cognitive symptoms in psychiatric disorders.

Cognitive dysfunction was first identified as an important clinical component of the symptomatology in schizophrenia and bipolar disorder (Kraepelin, 1919; Kaszniak et al., 1986; Akiskal and Akiskal, 2007; Konarski et al., 2007; Bondi et al., 2017; Raucher-Chéné et al., 2017; Bo et al., 2019). More recently, impairments in processing speed, executive function, memory, attention, and social cognition (Millan et al., 2012) have been highlighted in major depressive disorder (MDD) (Marazziti et al., 2010). Some of these deficits, such as processing speed, learning, and cognitive shifting, can be reversible upon remission from depression (Ahern and Semkovska, 2017). However, the majority of cognitive dysfunctions involving attention, inhibition, memory, and verbal fluency are acknowledged as more persistent (Årdal and Hammar, 2011; Rock et al., 2014; Shilyansky et al., 2016; Ahern and Semkovska, 2017). Because cognitive impairment in MDD is associated with reduced functioning and quality of life (McCall and Dunn, 2003) and MDD has a global point prevalence of 4.7% (Ferrari et al., 2012), improved treatment is needed to target persistent cognitive symptoms in MDD.

A plethora of studies have investigated associations between markers for the central serotonin (5-HT) system not only in MDD but also in anxiety disorders, obsessive compulsive disorder, substance use disorder, and schizophrenia as well as symptoms of aggression, suicidality, and psychopathic traits. Moreover, the central serotonin system has been implicated to be involved in normal cognitive function, including both general intellectual ability and specific cognitive domains. The serotonergic cell bodies are mainly localized in the raphe nuclei of the brainstem, from where their axons are projected throughout the brain. Serotonin is one of the most widely distributed neurotransmitters in the brain (Dahlström and Fuxe, 1964; Steinbusch, 1981) and exerts its effects via 14 receptor subtypes (5-HT_1_ – 5-HT_7_). The 5-HT_1A_ receptor has gained the most attention in psychiatric research, but studies of the 5-HT_1B_ receptor are emerging and point to its relevance for the pathophysiology and treatment of MDD (Murrough et al., 2011a, 2011b; Tiger et al., 2014, 2016, 2020). The 5-HT_1B_ receptor is an inhibitory G-protein coupled receptor with different functions depending on its location; stimulation of the presynaptic autoreceptor inhibits 5-HT release via a negative feedback system, while 5-HT_1B_ heteroreceptors are involved in modulation of the release of other neurotransmitters, such as dopamine and GABA (Barnes and Sharp, 1999; Tiger et al., 2018).

Pharmacological manipulations of the serotonin system have demonstrated effects on cognitive functions in healthy volunteers. Acute tryptophan depletion has been associated with deficits in decision-making (Rogers et al., 2003) and cognitive processing (Murphy et al., 2002). Administration of a 5-HT_1A_ partial agonist or a 5-HT_2A_ antagonist has been found to impair sustained attention (Wingen et al., 2007a) and verbal and spatial memory (Wingen et al., 2007b). Molecular neuroimaging studies in healthy individuals have shown positive correlations between serotonin transporter availability and general intellectual ability (Tseng et al., 2015) and performance in cognitive flexibility and logical reasoning (Madsen et al., 2011) as well as between 5-HT_1A_ availability and episodic memory (Penttilä et al., 2016). Also negative associations have been demonstrated between 5-HT_1A_ availability and memory (Yasuno et al., 2003) as well as a lack of correlation between 5-HT_1A_ availability and cognition (Borg et al., 2006, 2009).

Research on 5-HT_1B_ receptor availability and cognition has been scarce, but preclinical studies have suggested a modulatory role of the 5-HT_1B_ receptor on learning and memory-related behaviors (Wolff et al., 2003; Eriksson et al., 2008). Wolff and colleagues demonstrated enhanced learning performance in complex cognitive tasks in 5-HT_1B_ receptor knockout mice compared with wild-type mice (Wolff et al., 2003). In addition, the 5-HT_1B_ receptor agonist anpirtoline has been shown to impair performance in contextual memory in mice, while a 5-HT_1B_ antagonist improved performance (Eriksson et al., 2008).

In humans, a pharmacological challenge study of 2 5-HT_1B_ receptor agonists, sumatriptan and rizatriptan, reported enhanced speed and reduced accuracy after drug intake in healthy individuals (van der Post et al., 2002). Studies on vortioxetine, a serotonin reuptake inhibitor with 5-HT_1B_ agonist activity (Bang-Andersen et al., 2011; Yang et al., 2019), have indicated beneficial effects on psychomotor speed and executive control compared with selective serotonin reuptake inhibitors in MDD patients (Bennabi et al., 2019). A [^11^C]AZ10419369 positron emission tomography (PET) study in elderly control participants demonstrated a positive correlation between 5-HT_1B_ binding in total gray matter and creative ability measured with the Guilford’s alternative uses task when controlling for age (Varrone et al., 2015). Moreover, our previous pilot study showed an association between 5-HT_1B_ receptor availability in the dorsal brainstem (DBS) and visuospatial memory performance in the Rey’s complex figure test in healthy individuals without current or previous depressive episode (Tangen et al., 2017), which is also the focus of the present study.

In sum, although existing research supports involvement of the 5-HT system in cognitive functions, there are few direct studies of the association between 5-HT_1B_ receptor availability and cognition in humans, and the observations rarely have been replicated. Hence, the first aim of this study was to replicate the results from our previously published finding of an association between 5-HT_1B_ receptor availability in the DBS and visuospatial memory in healthy individuals (Tangen et al., 2017). A second aim was to explore putative relationships between 5-HT_1B_ receptor availability and other cognitive domains known to be affected in MDD in healthy participants.

## METHODS

### Procedure and Participants

Participants were recruited and examined at the PET Center at Karolinska Institutet, Stockholm, Sweden. The sample of the present study consisted of healthy individuals from previous studies investigating cognitive performance and 5-HT_1B_ receptor availability using PET and the radioligand [^11^C]AZ10419369 (Pierson et al., 2008). In all studies, participants were screened with a psychiatric interview, physical examination, and urine toxicology and, after inclusion, examined with cognitive assessment, structural magnetic resonance imaging (MRI), and PET examination with [^11^C]AZ10419369.

Out of 54 participants examined with PET, 43 were included in further analyses. The remaining 11 individuals were excluded due to no available cognitive data (10 individuals) or aberrant intensities within the MRI image resulting in failure of reliable segmentation (1 individual). In this study, the subjects were divided into 2 groups, where the replication sample (n = 34) was a subgroup of the exploratory sample (n = 43; see Statistics section for details). The PET data were previously published (Nord et al., 2013, 2014b; Tiger et al., 2014, 2016; Svensson et al., 2021). The studies were approved by the Ethics Committee of the Stockholm Region, the Radiation Safety Committee of the Karolinska Hospital, and, when applicable, the Medical Products Agency in Sweden. All studies were performed according to the guidelines of the Declaration of Helsinki, and all participants were given information about the study before giving written informed consent.

### Assessment of Cognitive Performance

Participants were examined with well-established cognitive tests associated with serotonin and MDD. Visuospatial memory was examined using Rey’s complex figure test, immediate recall (RCFT ir) and delayed recall (RCFT dr). Verbal fluency subtests letter production, category production, and flexibility were used to measure phonemic production, semantic production, and cognitive flexibility, respectively. Processing speed, task-switching ability, and cognitive flexibility were examined using Trailmaking test A and B with time as outcome measure. Attention and vigilance were assessed by Continuous performance test-II (CPT) with number of omission and commission errors, detectability (CPT d´) and reaction time (CPT rt) as outcome measures. Cognitive flexibility and reaction time were measured using Wisconsin card sorting test (WCST) with total errors, perseverative errors, and reaction time to positive feedback to unambiguous correct response (WCST rt) as outcome measures. Planning ability was assessed as time to complete the task in the Tower test from Delis Kaplan executive function system. General intellectual ability was estimated by Wechsler adult intelligence scale, third or fourth version, with subtests vocabulary and matrix reasoning and raw scores as outcome measure.

### Image Acquisition and Analysis

All participants were examined using MRI (3.0T, GE Healthcare). T2-weighted images were used for exclusion of structural pathology. T1-weighted images were used for delineating regions of interest (ROIs) and were co-registered with a summated PET image. SPM12 (Statistical Parametric Mapping, Wellcome Trust Centre for Neuroimaging, UK) was used to segment images into gray matter, white matter, and cerebrospinal fluid. [^11^C]AZ10419369 was synthesized as previously described (Pierson et al., 2008). All participants were examined using the ECAT HRRT (High Resolution Research Tomograph, Siemens Molecular Imaging) PET system (Wienhard et al., 2002). PET data were reconstructed using previously described methods (Varrone et al., 2009). Brain radioactivity was measured in a series of consecutive time-frames for 63 minutes (Nord et al., 2013, 2014b) or 93 minutes (Tiger et al., 2014, 2016; Svensson et al., 2021) depending on protocol. To enable pooling of data, only the first 63 minutes were used for quantification in the current analysis.

The original analyses for the 5 included studies used different ROI definition methods; the data were therefore reanalyzed for this study. ROIs were chosen based on the assumed clinical relevance for MDD and known distribution of the 5-HT_1B_ receptor according to the literature (Ruf and Bhagwagar, 2009; Tangen et al., 2017; Tiger et al., 2018). The following ROIs were included: whole brain cerebral gray matter, frontal cortex, orbitofrontal cortex (OFC), dorsolateral frontal cortex (DLFC), occipital cortex, anterior cingulate cortex (ACC), ventral striatum, hippocampus, amygdala, thalamus, insular cortex, limbic lobe, and DBS, a region including the raphe nuclei. The primary brain region of interest was total cerebral gray matter. The ROIs were automatically defined using the software package FreeSurfer (6.0.0, http://surfer.nmr.mgh.harvard.edu/), except for DBS, which was defined based on [^11^C]AZ10419369 PET template data (Veldman et al., 2021), and cerebral gray matter, which was defined by statistical parametric mapping segmentation. The cerebellar gray matter, having negligible 5-HT_1B_ receptor density (Varnäs et al., 2001), was used as reference region for quantification. To avoid spill-over from the occipital cortex, cerebrospinal fluid, and cerebellar vermis, the ROI for cerebellum was automatically adjusted as previously described (Matheson et al., 2017). Binding potential relative to the non-displaceable compartment was calculated using wavelet-aided parametric imaging (Cselényi et al., 2006).

### Statistics

Demographic data and descriptive statistics are presented in Table 1. The statistical evaluation was performed in 2 steps: replication analysis and exploratory analyses. Given a known negative correlation between age and cognitive performance as well as between age and 5-HT_1B_ receptor availability (Nord et al., 2014a), all analyses of the association between these variables were corrected for age (except when explicitly stated that such corrections were not performed).

**Table 1.** Demographic Data and Descriptive Statistics for the Replication Sample and the Exploratory Sample, Where the Replication Sample Is a Subsample of the Exploratory Sample

	Replication sample (total n = 34)			Exploratory sample (total n = 43)		
		Range	M (SD)	n	Range	M (SD)
Age	34	20–75	35.7 (15.1)	43	20–75	35.3 (14.2)
Gender	34	18 males; 16 females		43	23 males; 20 females	
Education	34	9–25	15.5 (3.5)	39	9–25	15.6 (3.4)
Handedness	34	31 right, 3 left		42	38 right, 4 left	
RCFT ir				39	9.5–34	22.5 (6.2)
RCFT dr	34	9.5–34	23.1 (6.3)	39	10–34	22.5 (6.4)
VF lp				43	20–73	44.3 (12.2)
VF cp				43	30–93	52.4 (12.4)
VF fl				26	10–21	14.4 (2.8)
CPT oms				35	0.0–30.0	3.2 (5.7)
CPT coms				35	1.0–31.0	11.6 (7.3)
CPT d´				35	0.1–1.9	0.8 (0.4)
CPT rt				33	302.4–623.8	408.8 (72.8)
TMT A				38	15–63	30.0 (11.0)
TMT B				38	34–283	80.3 (44.1)
ToT				17	205–1383	519.5 (315.6)
WCST te				29	7.0–94.0	34.2 (27.1)
WCST pe				29	4–56	17.4 (14.9)
WCST rt				29	0.86–4.9	2.2 (1.1)
WAIS vb				35	24–58	43 (8.1)
WAIS mr				17	11–25	20.3 (4.3)

Abbreviations: CPT coms, Continuous performance test-II, number of commissions; CPT d´, Continuous performance test-II, detectability (d´); CPT oms, Continuous performance test-II, number of omissions; CPT rt, Continuous performance test-II, reaction n time; Education n, years of education; M, mean; RCFT dr, Rey’s complex figure test, delayed recall; RCFT ir, Rey’s complex figure test, immediate recall; SD, standard deviation; TMT A, Trailmaking test A, seconds; TMT B, Trailmaking test B, seconds; ToT, D-KEFS Tower test, seconds; VF cp, Verbal fluency, category production; VF fl, Verbal fluency, flexibility; VF lp, Verbal fluency, letter production; WAIS mr, Wechsler adult intelligence scale, matrix reasoning, raw score; WCST pe, Wisconsin card sorting test, perseverative errors; WCST rt, Wisconsin card sorting test, reaction time; WCST te, Wisconsin card sorting test, total errors; WAIS vb, Wechsler adult intelligence scale, vocabulary, raw score.

The replication sample (n = 34) was a subgroup of the exploratory sample (n = 43) excluding participants (n = 5) from the previous pilot study (Tangen et al., 2017) and participants not examined with RCFT (n = 4). In the pilot study, we found a significant association between 5-HT_1B_ receptor availability in DBS and performance in RCFT dr (there named RCFT, delayed recognition) in the control group (Tangen et al., 2017) controlling for age. To replicate this finding, a partial correlation between 5-HT_1B_ receptor availability in DBS and RCFT dr with age as covariate was carried out.

Exploratory analyses of putative associations between 5-HT_1B_ receptor availability in whole brain gray matter as well as in specific ROIs and cognitive performance were performed with partial correlations controlling for age. Non-parametric tests were used for all variables as some of the cognitive test results were not normally distributed according to the Shapiro-Wilk test of normality (*P < *.05). The sample had a skewed age distribution (63% between 20 and 37 years of age), but as non-parametric analyses have been used, the results should not be affected by outliers. However, regression analyses examining dfBeta for outlying influential observations of combined age and 5-HT_1B_ receptor availability were performed. For comparison with the baseline data in our pilot study, non–age-adjusted Spearman’s correlations were also calculated in the exploratory sample (n = 43). Spearman’s correlations were calculated to investigate inter-regional 5-HT_1B_ receptor availability as well as associations between regional 5-HT_1B_ receptor availability and age and educational level, respectively. All tests were 2-tailed, except for the replication analysis, which was 1-tailed. For the confirmatory (i.e., replication) analysis, the significance level was set to *P *≤ .05. For the exploratory analyses, the strength of the association between variables was described using the following heuristic; a correlation of 0–0.19 was described as negligible, 0.2–0.39 as weak, 0.4–0.59 as moderate, 0.6–0.79 as strong, and 0.8–1 as very strong (Swinscow, 1997). Due to the exploratory nature, no correction for multiple comparisons was performed. Statistical analyses were performed using SPSS, version 26 for Mac.

## RESULTS

In the exploratory sample (n = 43), age-controlled weak to moderate correlations were demonstrated between number of commission errors in CPT and 5-HT_1B_ receptor availability in whole brain gray matter (*rho* = 0.39, *P* = .02) and specific brain regions, including frontal cortex (*rho* = 0.37, *P *=* *.03), DLFC (*rho *=* *0.36, *P *=* *.03), ACC (*rho *=* *0.42, *P *=* *.01), and limbic lobe (*rho *=* *0.35, *P *=* *.05). Also, weak to moderate correlations were found between CPT rt and 5-HT_1B_ availability in whole brain gray matter (*rho *=* *−0.41, *P *=* *.02), frontal cortex (*rho *=* *−0.37, *P *=* *.04), OFC (*rho *=* *−0.36, *P *=* *.04), DLFC (*rho *=* *−0.36, *P *=* *.05), and ACC (*rho *=* *−0.37, *P *=* *.04) as well as between time to complete the Tower test and 5-HT_1B_ receptor availability in thalamus (*rho *=* *0.56, *P *=* *.03; Table 2). Regression analyses investigating dfBeta revealed no statistical multivariate outlier. Correlation analyses not controlling for age between cognitive performance and 5-HT_1B_ receptor availability are specified in supplementary Table 1.

**Table 2.** Spearman’s Correlation (*rho*) Between Cognitive Performance and 5-HT_1B_ Availability in Brain Regions of Interest, Controlled for Age (n = 43)

	RCFT ir	RCFT dr	VF lp	VF cp	VF fl	CPT oms	CPT coms	CPT d´	CPT rt	TMT A	TMT B	ToT	WCST te	WCST pe	WCST rt
Gray matter	−0.13	−0.12	−0.03	0.14	0.10	0.21	0.39^*^	−0.26	−0.41^*^	0.04	−0.10	0.27	0.03	−0.04	−0.34^†^
FC	−0.24	−0.24	0.01	0.19	0.08	0.23	0.37^*^	−0.26	−0.37^*^	0.09	−0.05	0.09	0.23	0.16	−0.2
OFC	−0.26	−0.25	−0.02	0.14	0.17	0.16	0.31^†^	−0.21	−0.36^*^	0.13	−0.07	0.20	0.16	0.09	−0.23
DLFC	−0.22	−0.21	0.04	0.23	0.07	0.25	0.36^*^	−0.24	−0.36^*^	0.01	−0.06	0.13	0.18	0.12	−0.22
OC	−0.05	−0.03	−0.24	−0.14	−0.02	0.25	0.11	−0.06	−0.03	−0.02	−0.01	0.41	0.15	0.16	−0.25
ACC	−0.29^†^	−0.29^†^	0.13	0.20	0.08	0.21	0.42^*^	−0.32	−0.37^*^	0.01	−0.06	0.26	0.08	−0.02	−0.24
VSTR	−0.06	−0.06	−0.03	0.15	0.01	0.20	0.21	−0.16	−0.24	0.02	−0.13	0.05	0.04	−0.03	−0.07
HIP	−0.01	−0.01	−0.12	−0.11	−0.07	0.02	0.25	−0.22	−0.12	−0.04	−0.17	0.27	0.04	0.02	−0.04
Amygdala	−0.14	−0.12	0.01	0.13	0.30	0.12	0.05	−0.06	−0.12	−0.05	−0.28^†^	0.15	0.01	−0.09	−0.25
Thalamus	−0.02	−0.04	−0.08	−0.09	−0.01	0.01	−0.03	0.07	0.12	0.17	0.09	0.56^*^	0.12	0.14	−0.02
Insula	−0.20	−0.21	0.06	0.12	0.26	0.24	0.31^†^	−0.21	−0.32^†^	0.12	−0.08	0.11	0.15	0.03	−0.14
Limbic lobe	−0.11	−0.12	−0.01	0.15	0.06	0.25	0.35^*^	−0.26	−0.33^†^	0.05	−0.15	0.19	0.04	−0.04	−0.31
DBS	0.03	0.03	0.04	0.16	0.16	0.04	0.27	−0.15	−0.15	0.06	−0.07	0.44^†^	0.04	−0.04	−0.28

Abbreviations: ACC, anterior cingulated cortex; CPT coms, Continuous performance test-II, number of commissions; CPT d´, Continuous performance test-II, detectability (d´); CPT oms, Continuous performance test-II, number of omissions; CPT rt, Continuous performance test-II, reaction n time; DBS, dorsal brainstem; DLFC, dorsolateral frontal cortex; FC, frontal cortex; HIP, hippocampus; OC, occipital cortex; OFC, orbitofrontal cortex; RCFT dr, Rey’s complex figure test, delayed recall; RCFT ir, Rey’s complex figure test, immediate recall; TMT A, Trailmaking test A, seconds; TMT B, Trailmaking test B, seconds; ToT, D-KEFS Tower test, seconds; VF cp, Verbal fluency, category production; VF fl, Verbal fluency, flexibility; VF lp, Verbal fluency, letter production; VSTR, ventral striatum; WCST pe, Wisconsin n card sorting test, perseverative errors; WCST rt, Wisconsin n card sorting test, reaction n time; WCST te, Wisconsin n card sorting test, total errors.

*P < .05;

†*P < *.10.

In the replication sample (n = 34), Spearman’s partial correlation controlling for age, revealed no significant association between 5-HT_1B_ receptor availability in DBS and RCFT dr (*rho *=* *−0.08, *P *=* *.33).

There were positive and mostly very strong inter-regional correlations in 5-HT_1B_ availability among the 13 brain ROIs in the exploratory sample (n = 43), except between occipital cortex and hippocampus and between amygdala and thalamus (supplementary Table 2). For age effects, correlations were found between age and performance in RCFT ir (*rho *=* *−0.51, *P *≤ .001), RCFT dr (*rho *=* *−0.51, *P *≤ .001), CPT rt (*rho *=* *0.46, *P *=* *.01), and WCST rt (*rho *=* *0.53, *P *=* *.003). Age was negatively correlated to 5-HT_1B_ receptor availability in all brain regions (range in *rho* was −0.53 to −0.75), except in ventral striatum, hippocampus, and thalamus (supplementary Table 3). No correlations were found between educational level and cognitive test results or between educational level and 5-HT_1B_ receptor availability in any brain region.

## DISCUSSION

In the present study, we investigated the relationship between cognitive function and cerebral 5-HT_1B_ receptor availability in 43 healthy participants. In the exploratory analyses, weak to moderate correlations were observed between 5-HT_1B_ receptor availability in cortical regions and reaction time, error making, and planning ability. We could not replicate the positive association between 5-HT_1B_ receptor availability in DBS and visuospatial memory identified in our previous pilot study (Tangen et al., 2017).

In the exploratory analyses, weak to moderate positive correlations were found between number of errors made in CPT and 5-HT_1B_ receptor availability in whole brain gray matter, frontal cortex, DLFC, ACC, and the limbic lobe. Also, longer reaction time in CPT was associated with lower 5-HT_1B_ availability in whole brain gray matter, as well as the frontal cortex, OFC, DLFC, and ACC. Planning ability measured using the Tower test was positively correlated with 5-HT_1B_ receptor availability in thalamus. These results are in line with the reduced reaction time and increased number of errors found by van der Post and colleagues (2002) after administration of the 5-HT_1B_ agonists sumatriptan and rizatriptan to healthy control participants. The triptans were associated with a decreased reaction time and increased number of errors in response to a complex word recognition task requiring both speed and accuracy, but not in a simple reaction time task where only response to stimuli was required. In complex tasks such as the word recognition task in van der Post et al (2002), or the CPT test used in our study, the trade-off between fast reaction time and accuracy requires impulse inhibition. Thus, both stimulation of the 5-HT_1B_ receptor with agonists and high 5-HT_1B_ receptor availability could be associated with reduced impulse inhibition and increased impulsivity.

The hypothesis of an association between low central serotonin levels and high impulsivity and aggression is one of the longest standing in biological psychiatry (Hodge and Butcher, 1975; Coccaro, 1992; Duke et al., 2013). For cognitive impulsivity, the inability to inhibit a response rather than impulsiveness related to substance abuse and pathological gambling, global lowering of serotonin with tryptophan depletion has been shown to induce a more impulsive or disinhibited response style in a continuous performance test (Walderhaug et al., 2002) and to increase motor speed response to complex stimuli (Coull et al., 1995). Blockade of 5-HT_1A_ and 5-HT_2A_ receptors in individuals pre-treated with an selective serotonin reuptake inhibitor (SSRI) has been reported to impair attention but did not affect motor impulsivity in a stop-signal task (Wingen et al., 2007a). Involvement of the 5-HT_1B_ receptor in the serotonergic inhibitory control of responses is supported by findings of high impulsivity and aggression in 5-HT_1B_ receptor knock-out mice (Saudou et al., 1994; Rocha et al., 1998; Nautiyal et al., 2015). Findings from PET studies in human participants provide support for positive associations between high 5-HT_1B_ receptor availability and pathological gambling (Potenza et al., 2013), and high 5-HT_1B_ receptor availability has been reported for individuals with alcohol dependence (Hu et al., 2010) as well as emotional response inhibition (da Cunha-Bang et al., 2017) and violent behavior (da Cunha-Bang et al., 2018). Interindividual variability in 5-HT_1B_ receptor availability may partly represent differences in endogenous serotonin concentrations, either reflecting adaptive changes to synaptic serotonin levels or competition with endogenous transmitter at radioligand binding sites, consistent with an inverse relationship between endogenous serotonin levels and measures of impulsive behavior. Our observation of a negative correlation between 5-HT_1B_ availability and reaction time is therefore consistent with previous findings in PET studies of human participants and in line with the hypothesis of a relationship between low brain serotonin and impulsive action and a poorer ability to suppress responses.

In the pilot study (Tangen et al., 2017), an age-adjusted positive correlation between 5-HT_1B_ receptor availability in DBS and performance in the visuospatial memory test RCFT dr was found in the healthy control group. This association could not be replicated in this larger sample of healthy participants. The replication failure could be due to the use of different definitions of the DBS ROI. In the pilot study, the DBS was manually delineated, whereas the present study used an automated PET template-based approach to define the raphe nuclei. Moreover, it cannot be excluded that our replication failure may be due to the complex age effects on both 5-HT_1B_ receptor availability and cognitive performance described below, possibly confounding the associations between the two. Although this is the first replication attempt, our preliminary interpretation is that the pilot finding may be explained as a type I error.

The findings need to be interpreted within the context of the study limitations. First, a possible confounder of the results in the present study is the age effect on both 5-HT_1B_ receptor availability and cognitive test performance. Our group previously reported an average reduction of 8% for cortical 5-HT_1B_ binding potential relative to the non-displaceable compartment per decade (Nord et al., 2014a). Because age effects on most cognitive functions are well described in the literature, in particular for tasks requiring motor and processing speed, we controlled for age in our analyses of the relationship between 5-HT_1B_ receptor availability and cognitive test results. Nevertheless, it cannot be excluded that possible non-linear age effects may have confounded the result. Further replication studies are needed to confirm these findings, preferably in individuals from a more homogeneous age group. Second, our participants had a higher education level than the general population, with an average of more than 3 years of university. Although healthy individuals with higher educational level than the population is a general problem within medical research (Tambs et al., 2009) and our analyses did not reveal any education effects on 5-HT_1B_ receptor availability or cognitive results in this sample, the high education level should be taken into consideration when inferring the results on the general population. Lastly, in 4 participants, the time from PET examination to cognitive testing was nearly 1.5 years. Although the decline in 5-HT_1B_ receptor density and cognitive ability in the healthy population is expected to be minor within this time interval, an effect on the results cannot be ruled out.

In conclusion, although we failed to replicate the previously reported finding of a positive association between 5-HT_1B_ receptor availability in DBS and visuospatial memory, our observations support an association between 5-HT_1B_ receptor availability and number of errors made, reaction time, and planning ability. Though these results are exploratory and therefore should be interpreted with caution, they underscore previous findings in both animals and humans of the importance of the central serotonin system in impulsive behavior and planning ability.

## Supplementary Materials

Supplementary data are available at *International Journal of Neuropsychopharmacology (IJNPPY)* online.

pyac084_suppl_Supplementary_Tables

## Data Availability

All data underlying this article are available in the article and in its online supplementary material.

## Notes

This work was supported by the Swedish Research Council (523-2013-2982); Fonden för Psykisk Hälsa; a donation by Birgitta and Sten Westerberg; the Swedish Society of Medicine; Stiftelsen Söderström-Königska sjukhemmet; Hjärnfonden; Region Stockholm (ALF project Y00166, Clinical research appointment 2020-0735, and Clinical research appointment K2017-4579); The Stockholm Centre for Psychiatric Research and Education; and Karolinska Institutet.

Interest Statement: None.

## References

[CIT0001] Ahern E , Semkovska M (2017) Cognitive functioning in the first-episode of major depressive disorder: a systematic review and meta-analysis. Neuropsychology 31:52–72. 27732039 10.1037/neu0000319

[CIT0002] Akiskal HS , Akiskal KK (2007) A mixed state core for melancholia: an exploration in history, art and clinical science. Acta Psychiatr Scand Suppl 115:44–49. http://doi.wiley.com/10.1111/j.1600-0447.2007.00962.x. 10.1111/j.1600-0447.2007.00962.x 17280570

[CIT0003] Årdal G , Hammar A (2011) Is impairment in cognitive inhibition in the acute phase of major depression irreversible? Results from a 10-year follow-up study. Psychol Psychother 84:141–150. http://www.ncbi.nlm.nih.gov/pubmed/22903853. 22903853 10.1348/147608310X502328

[CIT0004] Bang-Andersen B , Ruhland T, Jørgensen M, Smith G, Frederiksen K, Jensen KG, Zhong H, Nielsen SM, Hogg S, Mørk A, Stensbøl TB (2011) Discovery of 1-[2-(2,4-dimethylphenylsulfanyl)phenyl]piperazine (Lu AA21004): a novel multimodal compound for the treatment of major depressive disorder. J Med Chem 54:3206–32021. http://www.ncbi.nlm.nih.gov/pubmed/21486038. 21486038 10.1021/jm101459g

[CIT0005] Barnes NM , Sharp T (1999) A review of central 5-HT receptors and their function. Neuropharmacology 38:1083–1152. http://www.ncbi.nlm.nih.gov/pubmed/10462127. 10462127 10.1016/s0028-3908(99)00010-6

[CIT0006] Bennabi D , Haffen E, Van Waes V (2019) Vortioxetine for cognitive enhancement in major depression: from animal models to clinical research. Front Psychiatry 10:771. http://www.ncbi.nlm.nih.gov/pubmed/31780961. 31780961 10.3389/fpsyt.2019.00771 PMC6851880

[CIT0007] Bo Q , Dong F, Li X, Li F, Li P, Yu H, He F, Zhang G, Wang Z, Ma X, Wang C (2019) Comparison of cognitive performance in bipolar disorder, major depressive disorder, unaffected first-degree relatives, and healthy controls. Psychiatry Clin Neurosci 73:70–76. 30393945 10.1111/pcn.12797

[CIT0008] Bondi MW , Edmonds EC, Salmon DP (2017) Alzheimer’s disease: past, present, and future. J Int Neuropsychol Soc 23:818–831. 29198280 10.1017/S135561771700100X PMC5830188

[CIT0009] Borg J , Andrée B, Lundberg J, Halldin C, Farde L (2006) Search for correlations between serotonin 5-HT1A receptor expression and cognitive functions - A strategy in translational psychopharmacology. Psychopharmacology 185:389–394. https://pubmed-ncbi-nlm-nih-gov.proxy.kib.ki.se/16541245/. 16541245 10.1007/s00213-006-0329-z

[CIT0010] Borg J , Henningsson S, Saijo T, Inoue M, Bah J, Westberg L, Lundberg J, Jovanovic H, Andrée B, Nordstrom A-L, Halldin C, Eriksson E, Farde L (2009) Serotonin transporter genotype is associated with cognitive performance but not regional 5-HT1A receptor binding in humans. Int J Neuropsychopharmacol 12:783. http://www.ncbi.nlm.nih.gov/pubmed/19126263. 19126263 10.1017/S1461145708009759

[CIT0011] Coccaro EF (1992) Impulsive aggression and central serotonergic system function in humans: an example of a dimensional brain-behavior relationship. Int Clin Psychopharmacol 7:3–12. https://pubmed-ncbi-nlm-nih-gov.proxy.kib.ki.se/1624755/. 10.1097/00004850-199200710-00001 1624755

[CIT0012] Coull JT , Sahakian BJ, Middleton HC, Young AH, Park SB, McShane RH, Cowen PJ, Robbins TW (1995) Differential effects of clonidine, haloperidol, diazepam and tryptophan depletion on focused attention and attentional search. Psychopharmacology 121:222–230. https://pubmed-ncbi-nlm-nih-gov.proxy.kib.ki.se/8545528/. 8545528 10.1007/BF02245633

[CIT0013] Cselényi Z , Olsson H, Halldin C, Gulyás B, Farde L (2006) A comparison of recent parametric neuroreceptor mapping approaches based on measurements with the high affinity PET radioligands [11C]FLB 457 and [11C]WAY 100635. Neuroimage 32:1690–1708. http://www.ncbi.nlm.nih.gov/pubmed/16859930. 16859930 10.1016/j.neuroimage.2006.02.053

[CIT0014] da Cunha-Bang S , Hjordt LV, Dam VH, Stenbæk DS, Sestoft D, Knudsen GM (2017) Anterior cingulate serotonin 1B receptor binding is associated with emotional response inhibition. J Psychiatr Res 92:199–204. https://pubmed-ncbi-nlm-nih-gov.proxy.kib.ki.se/28502766/. 28502766 10.1016/j.jpsychires.2017.05.003

[CIT0015] da Cunha-Bang S , Fisher PM, Hjordt LV, Perfalk E, Beliveau V, Holst K, Knudsen GM (2018) Men with high serotonin 1B receptor binding respond to provocations with heightened amygdala reactivity. Neuroimage 166:79–85. http://www.ncbi.nlm.nih.gov/pubmed/29061526. 29061526 10.1016/j.neuroimage.2017.10.032

[CIT0016] Dahlström A , Fuxe K (1964) A method for the demonstration of monoamine-containing nerve fibres in the central nervous system. Acta Physiol Scand 60:293–294. http://www.ncbi.nlm.nih.gov/pubmed/14131843. 14131843 10.1111/j.1748-1716.1964.tb02891.x

[CIT0017] Duke AA , Bègue L, Bell R, Eisenlohr-Moul T (2013) Revisiting the serotonin-aggression relation in humans: a meta-analysis. Psychol Bull 139:1148–1172. https://pubmed-ncbi-nlm-nih-gov.proxy.kib.ki.se/23379963/. 23379963 10.1037/a0031544 PMC3718863

[CIT0018] Eriksson TM , Madjid N, Elvander-Tottie E, Stiedl O, Svenningsson P, Ögren SO (2008) Blockade of 5-HT1B receptors facilitates contextual aversive learning in mice by disinhibition of cholinergic and glutamatergic neurotransmission. Neuropharmacology 54:1041–1050. https://linkinghub.elsevier.com/retrieve/pii/S0028390808000488. 18394658 10.1016/j.neuropharm.2008.02.007

[CIT0019] Ferrari AJ , Somerville AJ, Baxter AJ, Norman R, Patten SB, Vos T, Whiteford HA (2012) Global variation in the prevalence and incidence of major depressive disorder: a systematic review of the epidemiological literature. Psychol Med 43:471–481. http://www.ncbi.nlm.nih.gov/pubmed/22831756. 22831756 10.1017/S0033291712001511

[CIT0020] Hodge GK , Butcher LL (1975) Catecholamine correlates of isolation-induced aggression in mice. Eur J Pharmacol 31:81–93. https://pubmed-ncbi-nlm-nih-gov.proxy.kib.ki.se/1168580/. 1168580 10.1016/0014-2999(75)90081-3

[CIT0021] Hu J , Henry S, Gallezot J-D, Ropchan J, Neumaier JF, Potenza MN, Sinha R, Krystal JH, Huang Y, Ding Y-S, Carson RE, Neumeister A (2010) Serotonin 1B receptor imaging in alcohol dependence. Biol Psychiatry 67:800–803. 20172504 10.1016/j.biopsych.2009.12.028 PMC3112181

[CIT0022] Kaszniak AW , Wilson RS, Fox JH, Stebbins GT (1986) Cognitive assessment in Alzheimer’s disease: cross-sectional and longitudinal perspectives. Can J Neurol Sci 13:420–423. http://www.ncbi.nlm.nih.gov/pubmed/3491663. 3491663 10.1017/s0317167100037033

[CIT0023] Konarski JZ , McIntyre RS, Soczynska JK, Kennedy SH (2007) Neuroimaging approaches in mood disorders: technique and clinical implications. Ann Clin Psychiatry 19:265–277. http://www.ncbi.nlm.nih.gov/pubmed/18058284. 18058284 10.1080/10401230701653435

[CIT0024] Kraepelin E (1919) Dementia praecox and paraphrenia. Huntington, New York: Reprinted 1971 by Krieger Publishing Co.

[CIT0025] Madsen K , Erritzoe D, Mortensen EL, Gade A, Madsen J, Baaré W, Knudsen GM, Hasselbalch SG (2011) Cognitive function is related to fronto-striatal serotonin transporter levels--a brain PET study in young healthy subjects. Psychopharmacology 213:573–581. http://link.springer.com/10.1007/s00213-010-1926-4. 20623110 10.1007/s00213-010-1926-4

[CIT0026] Marazziti D , Consoli G, Picchetti M, Carlini M, Faravelli L (2010) Cognitive impairment in major depression. Eur J Pharmacol 626:83–86. http://www.ncbi.nlm.nih.gov/pubmed/19835870. 19835870 10.1016/j.ejphar.2009.08.046

[CIT0027] Matheson GJ , Stenkrona P, Cselényi Z, Plavén-Sigray P, Halldin C, Farde L, Cervenka S (2017) Reliability of volumetric and surface-based normalisation and smoothing techniques for PET analysis of the cortex: a test-retest analysis using [11C]SCH-23390. Neuroimage 155:344–353. http://www.ncbi.nlm.nih.gov/pubmed/28419852. 28419852 10.1016/j.neuroimage.2017.04.031

[CIT0028] McCall WV , Dunn AG (2003) Cognitive deficits are associated with functional impairment in severely depressed patients. Psychiatry Res 121:179–184. 14656452 10.1016/j.psychres.2003.09.003

[CIT0029] Millan MJ , et al (2012) Cognitive dysfunction in psychiatric disorders: characteristics, causes and the quest for improved therapy. Nat Rev Drug Discov 11:141–168. http://www.ncbi.nlm.nih.gov/pubmed/22293568. 22293568 10.1038/nrd3628

[CIT0030] Murphy F , Smith K, Cowen P, Robbins T, Sahakian B (2002) The effects of tryptophan depletion on cognitive and affective processing in healthy volunteers. Psychopharmacology (Berl) 163:42–53. http://www.ncbi.nlm.nih.gov/pubmed/12185399. 12185399 10.1007/s00213-002-1128-9

[CIT0031] Murrough JW , Czermak C, Henry S, Nabulsi N, Gallezot J-D, Gueorguieva R, Planeta-Wilson B, Krystal JH, Neumaier JF, Huang Y, Ding Y-S, Carson RE, Neumeister A (2011a) The effect of early trauma exposure on serotonin type 1B receptor expression revealed by reduced selective radioligand binding. Arch Gen Psychiatry 68:892. http://www.ncbi.nlm.nih.gov/pubmed/21893657. 21893657 10.1001/archgenpsychiatry.2011.91 PMC3244836

[CIT0032] Murrough JW , Henry S, Hu J, Gallezot J-D, Planeta-Wilson B, Neumaier JF, Neumeister A (2011b) Reduced ventral striatal/ventral pallidal serotonin1B receptor binding potential in major depressive disorder. Psychopharmacology 213:547–553. http://www.pubmedcentral.nih.gov/articlerender.fcgi?artid=3139174&tool=pmcentrez&rendertype=abstract. 20480149 10.1007/s00213-010-1881-0 PMC3139174

[CIT0033] Nautiyal KM , Tanaka KF, Barr MM, Tritschler L, Le Dantec Y, David DJ, Gardier AM, Blanco C, Hen R, Ahmari SE (2015) Distinct circuits underlie the effects of 5-HT1B receptors on aggression and impulsivity. Neuron 86:813–826. https://pubmed-ncbi-nlm-nih-gov.proxy.kib.ki.se/25892302/. 25892302 10.1016/j.neuron.2015.03.041 PMC4431594

[CIT0034] Nord M , Finnema SJ, Halldin C, Farde L (2013) Effect of a single dose of escitalopram on serotonin concentration in the non-human and human primate brain. Int J Neuropsychopharmacol 16:1577–1586. http://www.ncbi.nlm.nih.gov/pubmed/23351590. 23351590 10.1017/S1461145712001617

[CIT0035] Nord M , Cselenyi Z, Forsberg A, Rosenqvist G, Tiger M, Lundberg J, Varrone A, Farde L (2014a) Distinct regional age effects on [11C]AZ10419369 binding to 5-HT1B receptors in the human brain. Neuroimage 103:303–308. http://www.ncbi.nlm.nih.gov/pubmed/25255943. 25255943 10.1016/j.neuroimage.2014.09.040

[CIT0036] Nord M , Finnema SJ, Schain M, Halldin C, Farde L (2014b) Test-retest reliability of [11C]AZ10419369 binding to 5-HT(1B) receptors in human brain. Eur J Nucl Med Mol Imaging 41:301–307. http://www.ncbi.nlm.nih.gov/pubmed/24006152. 24006152 10.1007/s00259-013-2529-1

[CIT0037] Penttilä J , Hirvonen J, Tuominen L, Lumme V, Ilonen T, Någren K, Hietala J (2016) Verbal memory and 5-HT1A receptors in healthy volunteers – a PET study with [carbonyl-11C]WAY-100635. Eur Neuropsychopharmacol 26:570–577. http://www.ncbi.nlm.nih.gov/pubmed/26775837. 26775837 10.1016/j.euroneuro.2015.12.028

[CIT0038] Pierson ME , Andersson J, Nyberg S, McCarthy DJ, Finnema SJ, Varnäs K, Takano A, Karlsson P, Gulyás B, Medd AM, Lee C-M, Powell ME, Heys JR, Potts W, Seneca N, Mrzljak L, Farde L, Halldin C (2008) [11C]AZ10419369: a selective 5-HT1B receptor radioligand suitable for positron emission tomography (PET). Characterization in the primate brain. Neuroimage 41:1075–1085. http://www.ncbi.nlm.nih.gov/pubmed/18434202. 18434202 10.1016/j.neuroimage.2008.02.063

[CIT0039] Potenza MN , Balodis IM, Franco CA, Bullock S, Xu J, Chung T, Grant JE (2013) Neurobiological considerations in understanding behavioral treatments for pathological gambling. Psychol Addict Behav 27:380–392. https://pubmed-ncbi-nlm-nih-gov.proxy.kib.ki.se/23586456/. 23586456 10.1037/a0032389 PMC3700568

[CIT0040] Raucher-Chéné D , Achim AM, Kaladjian A, Besche-Richard C (2017) Verbal fluency in bipolar disorders: a systematic review and meta-analysis. J Affect Disord 207:359–366. http://www.ncbi.nlm.nih.gov/pubmed/27744224. 27744224 10.1016/j.jad.2016.09.039

[CIT0041] Rocha BA , Fumagalli F, Gainetdinov RR, Jones SR, Ator R, Giros B, Miller GW, Caron MG (1998) Cocaine self-administration in dopamine-transporter knockout mice. Nat Neurosci 1:132–137. https://pubmed-ncbi-nlm-nih-gov.proxy.kib.ki.se/10195128/. 10195128 10.1038/381

[CIT0042] Rock PL , Roiser JP, Riedel WJ, Blackwell AD (2014) Cognitive impairment in depression: a systematic review and meta-analysis. Psychol Med 44:2029–2040. http://www.ncbi.nlm.nih.gov/pubmed/24168753. 24168753 10.1017/S0033291713002535

[CIT0043] Rogers RD , Tunbridge EM, Bhagwagar Z, Drevets WC, Sahakian BJ, Carter CS (2003) Tryptophan depletion alters the decision-making of healthy volunteers through altered processing of reward cues. Neuropsychopharmacology 28:153–162. http://www.nature.com/articles/1300001. 12496952 10.1038/sj.npp.1300001

[CIT0044] Ruf BM , Bhagwagar Z (2009) The 5-HT1B receptor: a novel target for the pathophysiology of depression. Curr Drug Targets 10:1118–1138. http://www.eurekaselect.com/openurl/content.php?genre=article&issn=1389-4501&volume=10&issue=11&spage=1118. 19702551 10.2174/138945009789735192

[CIT0045] Saudou F , Amara DA, Dierich A, LeMeur M, Ramboz S, Segu L, Buhot MC, Hen R (1994) Enhanced aggressive behavior in mice lacking 5-HT1B receptor. Science (80-) 265:1875–1878. https://pubmed-ncbi-nlm-nih-gov.proxy.kib.ki.se/8091214/. 10.1126/science.8091214 8091214

[CIT0046] Shilyansky C , Williams LM, Gyurak A, Harris A, Usherwood T, Etkin A (2016) Effect of antidepressant treatment on cognitive impairments associated with depression: a randomised longitudinal study. Lancet Psychiatry 3:425–435. http://www.ncbi.nlm.nih.gov/pubmed/26995298. 26995298 10.1016/S2215-0366(16)00012-2 PMC4860142

[CIT0047] Steinbusch HW (1981) Distribution of serotonin-immunoreactivity in the central nervous system of the rat-cell bodies and terminals. Neuroscience 6:557–618. https://linkinghub.elsevier.com/retrieve/pii/0306452281901469. 7017455 10.1016/0306-4522(81)90146-9

[CIT0048] Svensson JE , Svanborg C, Plavén-Sigray P, Kaldo V, Halldin C, Schain M, Lundberg J (2021) Serotonin transporter availability increases in patients recovering from a depressive episode. Transl Psychiatry 11:264. https://pubmed-ncbi-nlm-nih-gov.proxy.kib.ki.se/33972499/. 33972499 10.1038/s41398-021-01376-w PMC8110529

[CIT0049] Swinscow TDV (1997) Statistics at Square One. 9th ed. BMJ Publ Gr. https://www.bmj.com/about-bmj/resources-readers/publications/statistics-square-one.

[CIT0050] Tambs K , Rønning T, Prescott CA, Kendler KS, Reichborn-Kjennerud T, Torgersen S, Harris JR (2009) The Norwegian Institute of Public Health twin study of mental health: examining recruitment and attrition bias. Twin Res Hum Genet 12:158–168. https://pubmed-ncbi-nlm-nih-gov.proxy.kib.ki.se/19335186/. 19335186 10.1375/twin.12.2.158 PMC2743739

[CIT0051] Tangen A , Borg J, Tiger M, Varnäs K, Sorjonen K, Lindefors N, Halldin C, Lundberg J (2017) Associations between cognition and serotonin receptor 1B binding in patients with major depressive disorder - a pilot study. Psychiatry Res 267:15–21. http://linkinghub.elsevier.com/retrieve/pii/S0925492716303663. 10.1016/j.pscychresns.2017.06.001 28688337

[CIT0052] Tiger M , Rück C, Forsberg A, Varrone A, Lindefors N, Halldin C, Farde L, Lundberg J (2014) Reduced 5-HT(1B) receptor binding in the dorsal brain stem after cognitive behavioural therapy of major depressive disorder. Psychiatry Res Neuroimaging 223:164–170. http://www.ncbi.nlm.nih.gov/pubmed/24916155. 10.1016/j.pscychresns.2014.05.011 24916155

[CIT0053] Tiger M , Farde L, Rück C, Varrone A, Forsberg A, Lindefors N, Halldin C, Lundberg J (2016) Low serotonin1B receptor binding potential in the anterior cingulate cortex in drug-free patients with recurrent major depressive disorder. Psychiatry Res Neuroimaging 253:36–42. http://www.sciencedirect.com/science/article/pii/S0925492715301426. 27269199 10.1016/j.pscychresns.2016.04.016

[CIT0054] Tiger M , Varnäs K, Okubo Y, Lundberg J (2018) The 5-HT1B receptor - a potential target for antidepressant treatment. Psychopharmacology 235:1317–1334. http://link.springer.com/10.1007/s00213-018-4872-1. 29546551 10.1007/s00213-018-4872-1 PMC5919989

[CIT0055] Tiger M , Veldman ER, Ekman C-J, Halldin C, Svenningsson P, Lundberg J (2020) A randomized placebo-controlled PET study of ketamine´s effect on serotonin1B receptor binding in patients with SSRI-resistant depression. Transl Psychiatry 10:159. http://www.nature.com/articles/s41398-020-0844-4. 32475989 10.1038/s41398-020-0844-4 PMC7261801

[CIT0056] Tseng PY , Lee IH, Chen KC, Chen PS, Chiu NT, Yao WJ, Chu CL, Yeh TL, Yang YK (2015) The correlation between mid-brain serotonin transporter availability and intelligence quotient in healthy volunteers. Eur Psychiatry 30:193–197. http://www.ncbi.nlm.nih.gov/pubmed/25447350. 25447350 10.1016/j.eurpsy.2014.09.001

[CIT0057] van der Post J , Schram MT, Schoemaker RC, Pieters MSM, Fuseau E, Pereira A, Baggen S, Cohen AF, van Gerven JMA (2002) CNS effects of sumatriptan and rizatriptan in healthy female volunteers. Cephalalgia 22:271–281. http://journals.sagepub.com/doi/10.1046/j.1468-2982.2002.00344.x. 12100089 10.1046/j.1468-2982.2002.00344.x

[CIT0058] Varnäs K , Hall H, Bonaventure P, Sedvall G (2001) Autoradiographic mapping of 5-HT1B and 5-HT1D receptors in the post mortem human brain using [3H]GR 125743. Brain Res 915:47–57. https://linkinghub.elsevier.com/retrieve/pii/S0006899301028232. 11578619 10.1016/s0006-8993(01)02823-2

[CIT0059] Varrone A , Sjöholm N, Eriksson L, Gulyás B, Halldin C, Farde L (2009) Advancement in PET quantification using 3D-OP-OSEM point spread function reconstruction with the HRRT. Eur J Nucl Med Mol Imaging 3610:1639–1650. https://link.springer.com/article/10.1007/s00259-009-1156-3. 10.1007/s00259-009-1156-3 19437012

[CIT0060] Varrone A , Svenningsson P, Marklund P, Fatouros-Bergman H, Forsberg A, Halldin C, Nilsson L-G, Farde L (2015) 5-HT1B receptor imaging and cognition: a positron emission tomography study in control subjects and Parkinson’s disease patients. Synapse 69:365–374. http://www.ncbi.nlm.nih.gov/pubmed/25914348. 25914348 10.1002/syn.21823

[CIT0061] Veldman ER , Varrone A, Varnäs K, Svedberg MM, Cselényi Z, Tiger M, Gulyás B, Halldin C, Lundberg J (2021) Serotonin 1B receptor density mapping of the human brainstem using positron emission tomography and autoradiography. J Cereb Blood Flow Metab 42:630–641. https://pubmed-ncbi-nlm-nih-gov.proxy.kib.ki.se/34644198/. 34644198 10.1177/0271678X211049185 PMC8943614

[CIT0062] Walderhaug E , Lunde H, Nordvik JE, Landrø NI, Refsum H, Magnusson A (2002) Lowering of serotonin by rapid tryptophan depletion increases impulsiveness in normal individuals. Psychopharmacology 164:385–391. https://pubmed-ncbi-nlm-nih-gov.proxy.kib.ki.se/12457268/. 12457268 10.1007/s00213-002-1238-4

[CIT0063] Wienhard K , Schmand M, Casey ME, Baker K, Bao J, Eriksson L, Jones WF, Knoess C, Lenox M, Lercher M, Luk P, Michel C, Reed JH, Richerzhagen N, Treffert J, Vollmar S, Young JW, Heiss WD, Nutt R (2002) The ECAT HRRT: performance and first clinical application of the new high resolution research tomograph. IEEE Trans Nucl Sci 49:104–110.

[CIT0064] Wingen M , Kuypers KPC, Ramaekers JG (2007a) The role of 5-HT1a and 5-HT2a receptors in attention and motor control: a mechanistic study in healthy volunteers. Psychopharmacology 190:391–400. http://www.ncbi.nlm.nih.gov/pubmed/17124621. 17124621 10.1007/s00213-006-0614-x

[CIT0065] Wingen M , Kuypers KPC, Ramaekers JG (2007b) Selective verbal and spatial memory impairment after 5-HT1A and 5-HT2A receptor blockade in healthy volunteers pre-treated with an SSRI. J Psychopharmacol 21:477–485. http://www.ncbi.nlm.nih.gov/pubmed/17092965. 17092965 10.1177/0269881106072506

[CIT0066] Wolff M , Savova M, Malleret G, Hen R, Segu L, Buhot M-C (2003) Serotonin 1B knockout mice exhibit a task-dependent selective learning facilitation. Neurosci Lett 338:1–4. http://www.ncbi.nlm.nih.gov/pubmed/12565126. 12565126 10.1016/s0304-3940(02)01339-3

[CIT0067] Yang K-C , Stepanov V, Amini N, Martinsson S, Takano A, Bundgaard C, Bang-Andersen B, Sanchez C, Halldin C, Farde L, Finnema SJ (2019) Effect of clinically relevant doses of vortioxetine and citalopram on serotonergic PET markers in the nonhuman primate brain. Neuropsychopharmacology 44:1706–1713. http://www.nature.com/articles/s41386-019-0442-4. 31216565 10.1038/s41386-019-0442-4 PMC6784989

[CIT0068] Yasuno F , Suhara T, Nakayama T, Ichimiya T, Okubo Y, Takano A, Ando T, Inoue M, Maeda J, Suzuki K (2003) Inhibitory effect of hippocampal 5-HT1A receptors on human explicit memory. Am J Psychiatry 160:334–340. https://pubmed-ncbi-nlm-nih-gov.proxy.kib.ki.se/12562581/. 12562581 10.1176/appi.ajp.160.2.334

