# Supplementary material for: Associations Between Cognition and Serotonin 1B Receptor Availability in Healthy Volunteers: A [11C]AZ10419369 Positron Emission Tomography Study
Source: Int J Neuropsychopharmacol. 2022 Dec 27;26(4):241–8. doi: 10.1093/ijnp/pyac084 (PMC10109060; doi:10.1093/ijnp/pyac084)
Supplement: pyac084_suppl_Supplementary_Tables [file pyac084_suppl_supplementary_tables.docx]

| Supplementary Table S1. Spearman´s correlation (*rho*) between cognitive performance and 5-HT_1B_ availability in brain regions of interest, not controlled for age (*n*= 43). | | | | | | | | | | | | | | | |
| --- | --- | --- | --- | --- | --- | --- | --- | --- | --- | --- | --- | --- | --- | --- | --- |
|  | RCFT ir | RCFT dr | VF lp | VF cp | VF fl | CPT oms | CPT coms | CPT d´ | CPT rt | TMT A | TMT B | ToT | WCST te | WCST pe | WCST rt |
| GM | 0.29^†^ | 0.30^†^ | 0.14 | -0.01 | 0.16 | 0.03 | 0.33^†^ | -0.23 | -0.59^*^ | -0.08 | -0.26 | 0.05 | -0.20 | -0.27 | -0.56^*^ |
| FC | 0.20 | 0.20 | 0.16 | 0.04 | 0.15 | 0.07 | 0.34^*^ | -0.24 | -0.55^*^ | -0.04 | -0.21 | -0.04 | -0.03 | -0.09 | -0.44^*^ |
| OFC | 0.22 | 0.23 | 0.15 | -0.01 | 0.21 | 0.01 | 0.28 | -0.21 | -0.55^*^ | -0.03 | -0.24 | 0.02 | -0.11 | -0.17 | -0.49^*^ |
| DLFC | 0.20 | 0.21 | 0.18 | 0.07 | 0.13 | 0.09 | 0.33^†^ | -0.23 | -0.54^*^ | -0.10 | -0.22 | 0.01 | -0.07 | -0.12 | -0.46^*^ |
| OC | 0.37^*^ | 0.38^*^ | 0.01 | -0.20 | 0.09 | 0.03 | 0.14 | -0.1 | -0.37^*^ | -0.13 | -0.21 | 0.08 | -0.20 | -0.21 | -0.55^*^ |
| ACC | 0.09 | 0.09 | 0.24 | 0.07 | 0.14 | 0.05 | 0.37^*^ | -0.29^†^ | -0.54^*^ | -0.08 | -0.20 | 0.10 | -0.14 | -0.22 | -0.47^*^ |
| VSTR | 0.11 | 0.11 | 0.04 | 0.10 | 0.04 | 0.11 | 0.22 | -0.18 | -0.37^*^ | -0.03 | -0.20 | -0.01 | -0.11 | -0.18 | -0.27 |
| HIP | 0.11 | 0.11 | -0.05 | -0.15 | -0.01 | -0.03 | 0.26 | -0.24 | -0.27 | -0.08 | -0.22 | 0.19 | -0.03 | -0.05 | -0.13 |
| Amygdala | 0.17 | 0.19 | 0.13 | 0.03 | 0.32 | -0.01 | 0.09 | -0.09 | -0.36^*^ | -0.13 | -0.37^*^ | -0.01 | -0.21 | -0.29 | -0.49^*^ |
| Thalamus | 0.14 | 0.12 | -0.03 | -0.12 | -0.01 | 0 | -0.03 | 0.07 | 0.08 | 0.11 | -0.01 | 0.47^†^ | 0.09 | 0.11 | -0.05 |
| Insula | 0.23 | 0.23 | 0.21 | -0.02 | 0.29 | 0.06 | 0.28 | -0.21 | -0.52^*^ | -0.02 | -0.24 | -0.02 | -0.10 | -0.20 | -0.41^*^ |
| LL | 0.29^†^ | 0.29^†^ | 0.15 | 0.01 | 0.14 | 0.06 | 0.30^†^ | -0.24 | -0.53^*^ | -0.08 | -0.29^†^ | 0 | -0.19 | -0.26 | -0.54^*^ |
| DBS | 0.35^*^ | 0.35^*^ | 0.18 | 0.02 | 0.21 | -0.07 | 0.26 | -0.17 | -0.41^*^ | -0.05 | -0.22 | 0.11 | -0.16 | -0.22 | -0.48^*^ |
| Note. RCFT ir= Rey´s complex figure test, immediate recall; RCFT dr= Rey´s complex figure test, delayed recall; VF lp= Verbal fluency, letter production; VF cp= Verbal fluency, category production; VF fl= Verbal fluency, flexibility; CPT oms= Continuous performance test-II, number of omissions; CPT coms= Continuous performance test-II, number of commissions; CPT d´= Continuous performance test-II, detectability (d´); CPT rt= Continuous performance test-II, reaction time; TMT A= Trailmaking test A, seconds; TMT B= Trailmaking test B, seconds; ToT= D-KEFS Tower test, seconds; WCST te= Wisconsin card sorting test, total errors; WCST pe= Wisconsin card sorting test, perseverative errors; WCST rt= Wisconsin card sorting test, reaction time; GM= gray matter; FC= frontal cortex; OFC= orbitofrontal cortex; DLFC= dorsolateral frontal cortex; OC= occipital cortex; ACC= anterior cingulated cortex; VSTR= ventral striatum; HIP= hippocampus; LL= limbic lobe; DBS= dorsal brainstem; *= p< 0.05; †= p< 0.10. | | | | | | | | | | | | | | | |

| Supplementary Table S2. Spearman´s correlations (*rho*) between 5-HT_1B_ availability in brain regions of interest (*n*= 43). | | | | | | | | | | | | |
| --- | --- | --- | --- | --- | --- | --- | --- | --- | --- | --- | --- | --- |
|  | GM | Neocortex | | | | Subcortical regions | | | | | | |
|  |  | FC | OFC | DLFC | OC | ACC | VSTR | HIP | AMG | THA | INS | LL |
| FC | 0.96^***^ |  |  |  |  |  |  |  |  |  |  |  |
| OFC | 0.94^***^ | 0.96^***^ |  |  |  |  |  |  |  |  |  |  |
| DLFC | 0.96^***^ | 0.99^***^ | 0.93^***^ |  |  |  |  |  |  |  |  |  |
| OC | 0.85^***^ | 0.81^***^ | 0.80^***^ | 0.82^***^ |  |  |  |  |  |  |  |  |
| ACC | 0.93^***^ | 0.92^***^ | 0.87^***^ | 0.92^***^ | 0.74^***^ |  |  |  |  |  |  |  |
| VSTR | 0.65^***^ | 0.65^***^ | 0.64^***^ | 0.62^***^ | 0.58^***^ | 0.72^***^ |  |  |  |  |  |  |
| HIP | 0.44^**^ | 0.36^*^ | 0.40^**^ | 0.34^*^ | 0.26 | 0.36^*^ | 0.43^**^ |  |  |  |  |  |
| AMG | 0.68^***^ | 0.60^***^ | 0.69^***^ | 0.58^***^ | 0.55^***^ | 0.66^***^ | 0.60^***^ | 0.50^***^ |  |  |  |  |
| THA | 0.40^**^ | 0.36^*^ | 0.39^**^ | 0.35^*^ | 0.41^**^ | 0.34^*^ | 0.43^**^ | 0.39^**^ | 0.26 |  |  |  |
| INS | 0.93^***^ | 0.91^***^ | 0.92^***^ | 0.89^***^ | 0.77^***^ | 0.91^***^ | 0.68^***^ | 0.48^***^ | 0.73^***^ | 0.48^***^ |  |  |
| LL | 0.95^***^ | 0.92^***^ | 0.90^***^ | 0.91^***^ | 0.80^***^ | 0.93^***^ | 0.74^***^ | 0.51^***^ | 0.71^***^ | 0.49^***^ | 0.93^***^ |  |
| DBS | 0.84^***^ | 0.79^***^ | 0.84^***^ | 0.78^***^ | 0.68^***^ | 0.75^***^ | 0.53^***^ | 0.52^***^ | 0.64^***^ | 0.58^***^ | 0.85^***^ | 0.85^***^ |
| Note. GM= gray matter; FC= frontal cortex; OFC= orbitofrontal cortex; DLFC= dorsolateral frontal cortex; OC= occipital cortex; ACC= anterior cingulate cortex; VSTR= ventral striatum; HIP= hippocampus; AMG= amygdala; THA= thalamus; INS= insula; LL= limbic lobe; DBS= dorsal brainstem; ^***^= *p*< 0.000; **^**^**= *p*< 0.01; **^*^**= *p*< 0.05. | | | | | | | | | | | | |

| Supplementary Table S3. Spearman´s correlation (*rho*) between age and 5-HT_1B_ availability in brain regions of interest (*n*= 43). | | | | | | | | | | | | | |
| --- | --- | --- | --- | --- | --- | --- | --- | --- | --- | --- | --- | --- | --- |
|  | GM | FC | OFC | DLFC | OC | ACC | VSTR | HIP | AMG | THA | INS | LL | DBS |
| Age | –0.73^***^ | –0.69^***^ | –0.73^***^ | –0.68^***^ | –0.75^***^ | –0.60^***^ | –0.28**^†^** | –0.29**^†^** | –0.53^***^ | –0.20 | –0.72^***^ | –0.70^***^ | –0.67^***^ |
| Note. GM= gray matter; FC= frontal cortex; OFC= orbitofrontal cortex; DLFC= dorsolateral frontal cortex; OC= occipital cortex; ACC= anterior cingulate cortex; VSTR= ventral striatum; HIP= hippocampus; AMG= amygdala; THA= thalamus; INS= insula; LL= limbic lobe; DBS= dorsal brainstem; ^***^= *p*< 0.001; **^†^**= *p*< 0.1. | | | | | | | | | | | | | |
